# Caloric vestibular stimulation in aphasic syndrome

**DOI:** 10.3389/fnint.2013.00099

**Published:** 2013-12-23

**Authors:** David Wilkinson, Rachael Morris, William Milberg, Mohamed Sakel

**Affiliations:** ^1^School of Psychology, University of KentCanterbury, Kent, UK; ^2^Department of Psychiatry, Harvard Medical SchoolBoston, MA, USA; ^3^East Kent Neuro-Rehabilitation Service, East Kent Hospitals University NHS Foundation TrustKent, UK

**Keywords:** neuro-stimulation, stroke, language, communication, rehabilitation

## Abstract

Caloric vestibular stimulation (CVS) is commonly used to diagnose brainstem disorder but its therapeutic application is much less established. Based on the finding that CVS increases blood flow to brain structures associated with language and communication, we assessed whether the procedure has potential to relieve symptoms of post-stroke aphasia. Three participants, each presenting with chronic, unilateral lesions to the left hemisphere, were administered daily CVS for four consecutive weeks. Relative to their pre-treatment baseline scores, two of the three participants showed significant improvement on both picture and responsive naming at immediate and 1-week follow-up. One of these participants also showed improved sentence repetition, and another showed improved auditory word discrimination. No adverse reactions were reported. These data provide the first, albeit tentative, evidence that CVS may relieve expressive and receptive symptoms of aphasia. A larger, sham-controlled study is now needed to further assess efficacy.

## INTRODUCTION

Aphasia is a language disorder most commonly caused by stroke to the left cerebral hemisphere in right-handed adults (Hamilton et al., 2011). The condition often disrupts comprehension, speech, reading, and writing, and impacts general rehabilitative outcome (Code and Herrmann, 2003; Paolucci et al., 2005; Hilari, 2011). Aphasia affects approximately 38% of patients who suffer a left hemisphere stroke, persisting in approximately 40–60% of cases (Meinzer et al., 2005). Unfortunately, the conventional treatment of speech and language therapy is often not effective, especially in chronic cases (Robey, 1994; Berthier, 2005; Kelly et al., 2010; Brady et al., 2012). Preliminary success in relieving certain symptoms of aphasia has, however, been achieved using transcranial direct current stimulation and transcranial magnetic stimulation (e.g., Naeser et al., 2005b; You et al., 2011). Here we investigated the potential efficacy of another form of non-invasive neuro-modulation, caloric vestibular stimulation (CVS).

Caloric vestibular stimulation involves the transmission of either warm or cool temperature, usually via water or air, from the external ear canal to the vestibular organs located in the adjacent labyrinth. These temperatures alter the density of endolymphatic fluid within the semi-circular canals and otolith organs, which in turn modulates the firing rates of vestibular hair cells. The resulting change in vestibular nerve activity is interpreted by the brain as a natural head movement, and increases blood flow across many cortical regions including language areas 44/45 (Broca’s) and 22 (Wernicke’s) (Fasold et al., 2002; Dieterich et al., 2003). These flow changes may be important because there is evidence from brain-injured patients that they correlate with spontaneous improvements in repetition and comprehension (Muira et al., 1999; Heiss and Thiel, 2006; Hamilton et al., 2011). Vestibular stimulation also modulates the release of glutamate (Horii et al., 1994; Holstein et al., 2012), nor-adrenaline (Nishiike et al., 2001), serotonin (Ma et al., 2007), and acetylcholine (Horii et al., 1994), all of which have been implicated in cognitive function and recovery (Klein and Albert, 2004).

A small number of studies have monitored language ability during CVS. Magrun et al. (1981) reported spontaneous improvement in the speech of developmentally delayed children following 5, 10 min CVS sessions. Schiff and Pulver (1999) reported a case of acute aphasia in whom brief language improvement occurred shortly after a single session of CVS of unspecified duration. This patient presented with severe expressive difficulties, but following CVS was briefly able to produce full sentences using appropriate emotion and intonation. By contrast, Vallar et al. (1995) described another case in which CVS did not improve language. However, this observation was based on 1 min sessions of CVS which are much shorter than the durations used in neurostimulation studies that have reported favorable language outcomes (Naeser et al., 2005b; Baker et al., 2010). Recently, for example, Barwood et al. (2012) applied low frequency repetitive transcranial magnetic stimulation (rTMS) to the right homolog of the pars triangularis of Broca’s area (BA 45) for 20 min per day and found a subsequent improvement in picture naming, repetition, and auditory comprehension (for similar outcomes using rTMS, see Naeser et al., 2005a,b; Hamilton et al., 2010). In a similar vein, You et al. (2011) showed improved auditory comprehension and spontaneous speech following repeated 30 min administrations of cathodal transcranial direct current stimulation (tDCS) to the right homolog of Wernicke’s area (for a similar outcome using tDCS, see Kang et al., 2011).

Compared to CVS, however, TMS and tDCS have several shortcomings that limit their rehabilitative potential. Firstly, both techniques involve the stimulation of a specific brain area which requires *a priori* knowledge of where to position the magnetic coil/electrodes. This can be particularly difficult when working with stroke patients whose functional anatomy is often altered by the presence of a lesion (Bolognini et al., 2009). TMS is also associated with an increased risk of seizure and is difficult to miniaturize, while tDCS is contraindicated for individuals with electronic implants or certain types of metal plates (Rossi et al., 2009).

The traditional method of CVS involves irrigating the external ear canal with ice-cold water. It is difficult to control the temperature and rate of flow during irrigation, and perhaps, more important, the presence of cold water in the external ear canal induces vertigo, a strong horizontal nystagmus and nausea. Advances in biomedical engineering have, however, led to the development of a solid state device that can warm or cool the external ear canal via a small thermal-electric probe. The temperature can be easily maintained a few degrees above 15°C which is the approximate point at which the vestibular nerves reach asymptote (Reker, 1977). Importantly, this temperature range is not associated with nausea, marked nystagmus or vertigo. An added benefit of solid-state devices is that they can be made highly portable and easy to use, so are suitable for home-based, self-administration.

The aim of the current study was to seek preliminary evidence for the hypothesis that the administration of CVS can result in measurable improvements in language function in patients with aphasia. We recruited three individuals in the sub-acute/chronic phase (i.e., >6 months post-onset) of stroke who were suffering from receptive and expressive aphasia. All had shown either little or no improvement in language ability within the preceding 3 months. Each individual received 20 days of CVS, with each daily session lasting 20 mins. Language and communication was assessed using subtests of the Boston Diagnostic Aphasia Examination (BDAE; Goodglass et al., 2001) which were administered across two baseline sessions, on the final day of stimulation, and then at 1 and 4 weeks post-stimulation. We did not include a sham condition because we felt it unreasonable at such an early stage of study to ask brain-injured patients to travel to the clinic every day for 4 weeks with no realistic prospect of gain. Given the failure of many speech and language rehabilitation programs, we felt it more appropriate to first establish whether any of our participants actually showed improvement, and for how long. Once the wash-out period had been estimated, a subsequent study could implement a cross-over design that exposed all participants to appropriately spaced active and sham treatments.

## CASE HISTORIES

Three right-handed aphasic individuals were recruited via physician referral from the Kent and Canterbury Hospital, East Kent Hospitals University NHS Foundation Trust. All participants had suffered an ischemic stroke at least 6 months prior to study enrolment, and had received speech and language therapy during the acute phase. Given the exploratory nature of the study, participants were only excluded if they had not suffered a left, unilateral stroke, had a significant history of neurological or medical illness, or presented with inner ear pathology or hearing difficulties.

**FIGURE 1:**
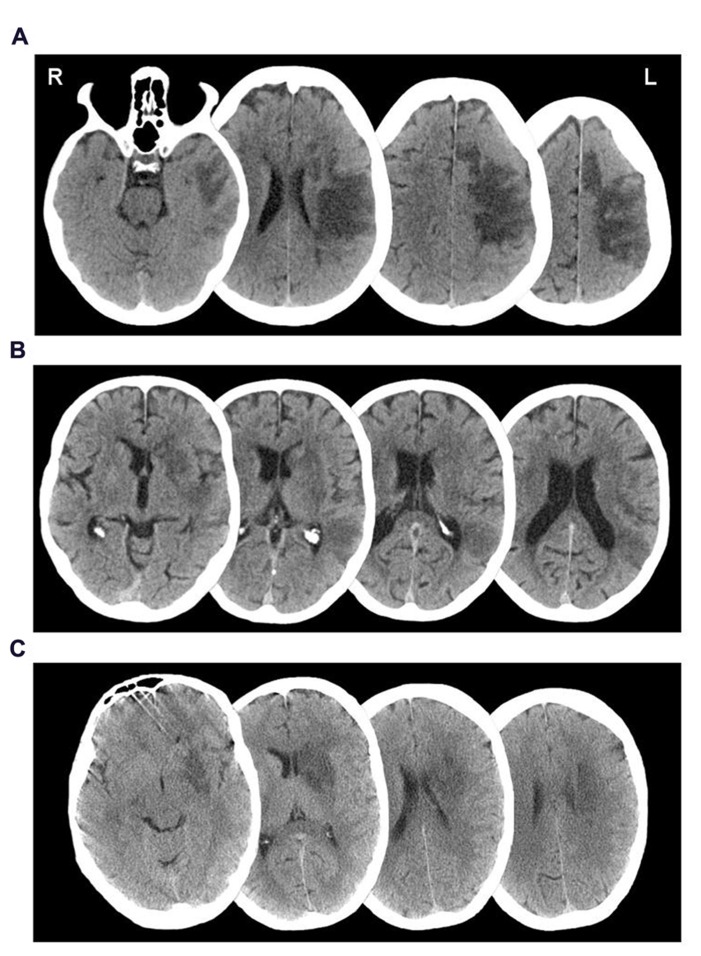
**Axial CT imageshowing lesion distributions of participants (A) 001,(B) 002, and **(C)** 003**.

Participant 001, a 62-year-old female, was admitted to hospital following a left middle cerebral artery (MCA) infarct, 11 months prior to study enrolment. CT investigation at admission indicated a large area of ischemic damage within the territory of the left MCA, with additional involvement of the caudate nucleus (see **Figure 1A**). A supplementary MRI investigation 4 months post-injury showed evidence of a prior hemorrhage most noticeably affecting segments M2 and M5 of the left MCA and the lenticular nucleus. The results were suggestive of persisting stenosis in the left sylvian artery at segment M2 of the MCA.

Speech therapy reports completed soon after admission indicated that 001 showed severe global aphasia affecting all language modalities. At the time of study enrolment, 001 had shown some improvement in receptive communication, though spoken output was limited to single words and short automatic phrases. Repetition was also moderately impaired; 001 was able to repeat single words well but could not manage short sentences. Progress in speech and physiotherapy had been limited, largely due to 001’s complex presentation that also involved low mood, anxiety, and tearfulness.

At the time of study enrolment, 001 was living semi-independently in her own home with the support of her husband and carers. Her rehabilitation physician advised that her speech and language ability had been stable for several months. She was still unable to transfer independently and scored 3/5 on the Modified Ashworth Scale (MAS; Bohannon and Smith, 1987) for the right flexor digitorum profundus and flexor digitorum superficialis. Perceptual assessment revealed a mild, right-sided visual hemi-spatial neglect, as assessed by subtests of the Behavioral Inattention Test (Wilson et al., 1987). She also showed a constructional apraxia, scoring within the moderate-severely impaired range for both the copying accuracy and planning elements of the Rey–Osterrieth Complex Figure task (Stern et al., 1999), and failing to progress past the initial trial of the WAIS-R Block Design subtest (Wechsler, 1981).

Participant 002, a 70-year-old male, was admitted to hospital 8 months prior to study enrolment, having suffered a left MCA infarct. A CT investigation revealed low attenuation in the left basal ganglia, left parietal, temporal and frontal lobes, plus sulcal effacement and compression of the anterior horn of the left lateral ventricle (see **Figure 1B**). A carotid Doppler test carried out a month later indicated complete occlusion of the left internal carotid artery.

Participant 002 presented with right hemiplegia, reduced coordination of the right upper limb and reduced balance. At the time of study enrolment, 002 was able to walk short distances with supervision and the use of a walking stick, though required the use of a wheelchair when outdoors and had difficulty mobilizing due to distractions. He showed difficulty with motor planning and sequencing and over-activity into flexion in his right upper limb during functional tasks, though this could be overcome by prompting. Poor scapular control and awareness limited the active range of his right glenohumeral joint.

Speech therapy prior to study enrolment indicated severe expressive aphasia and apraxia of speech. Spontaneous spoken output was largely unintelligible. Repetition was also significantly impaired, even at single word level. Apraxic difficulties were evident in attempts to copy lip and tongue movements and repeat sounds. In addition, 002 presented with moderate receptive difficulties. Participant 002 had regularly expressed frustration and low mood at difficulties during speech therapy sessions. At the time of study enrolment, 002 was living semi-independently at home with the support of his wife and carers. His rehabilitation physician advised that his speech and language ability had been stable for several months. On the Rey–Osterrieth Complex Figure task, participant 002 scored within the low-average range for copying accuracy and below average/mildly impaired for the planning element (Stern et al., 1999). He showed moderate to severe impairment on the WAIS-R Block Design subtest (Wechsler, 1981).

Participant 003, a-51-year old male, was admitted to hospital upon sudden onset of aphasia and right-sided weakness, 22 months prior to study enrolment. A CT scan taken upon admission revealed a low density area in the deep white matter of the left posterior frontal lobe, lateral to the anterior horn of the left ventricle (see **Figure 1C**). An additional MRI scan a week later indicated a hyperdense signal in the left temporal lobe, left basal ganglia, left caudate nucleus, and internal capsule extending into the left semiovale. Magnetic resonance angiography (MRA) indicated complete occlusion of the left MCA and left internal carotid.

Speech assessment prior to discharge from hospital highlighted severe expressive and receptive aphasia. Follow up with the community speech therapist suggested that his comprehension difficulties had mostly resolved following discharge, but spoken output remained limited to occasional words and short phrases. Participant 003 also presented with a mild apraxia of speech. Participant 003’s speech presentation had remained stable up until 6 weeks prior to study enrolment, when a period of modest, spontaneous recovery occurred. During this time participant 003 began to say more words and sentences, though overall verbal output still remained limited. 003 further presented with a mild right-sided weakness resulting in restricted upper limb movement and reduced fine motor skills. There was muscle weakness of the right lower limb, although he was completely mobile. He scored 5/5 on the Functional Ambulation Categories (Holden et al., 1984), 4/5 on the MRC muscle power testing (Medical Research Council, 1986), and 1/5 on the MAS (Bohannon and Smith, 1987) for his upper right limb, and 40/40 on the Modified Rivermead Mobility Index (Lennon and Johnson, 2000). Tests of visual construction and lateralised attention were not performed because, unlike participants 001 and 002, there was no mention of these capacities at hospital discharge. At the time of study enrolment, Participant 003 was living largely independently at home with his wife.

## LANGUAGE OUTCOME MEASURES

Participants were assessed on various sub-tests of the BDAE third Edition (Goodglass et al., 2001) – see **Table 1**.

**Table 1:** BDAE outcome measures.

	Sub-test	Description
Naming	BNT short form	Participants are presented with the following pictures and asked to name them: house, comb, toothbrush, octopus, bench, volcano, canoe, beaver, cactus, hammock, stethoscope, unicorn, tripod, sphinx, and palette.
	Responsive naming	Participants are required to answer questions such as “What color is grass?” and “What do we tell the time with?” Two points are given for responses within 5 s, one point for over 5 s.
	Naming in categories	Participants are required to name pictures organized into categories of colors, animals, actions, and tools/implements.
Connected speech	Cookie theft description	Participants are asked to describe a complex scene presented in picture format. They are scored on the longest phrase produced.
Repetition	Single words, non-sense words, sentences	Participants are asked to repeat single words, non-sense words and sentences.
Auditory comprehension	Basic word descrimination	Participants are asked to point to a verbally presented object from a selection of pictures.
	Commands	Participants are asked to follow instructions to, for example, “point to the ceiling and then to the floor.” One point is awarded for each component of the command that is completed correctly.
	Complex ideational material	Participants are required to answer yes/no questions about a verbally presented paragraph. Each question consists of two yes/no questions. Both must be answered correctly for one point.

## TEST SCHEDULE

Favorable ethical approval was gained from the University of Kent Psychology Research Ethics Committee, and all participants gave written, informed consent prior to study.

Baseline assessments were administered 10 and 3 days prior to stimulation to gage pre-treatment language ability. CVS was performed from Monday to Friday for the next 4 weeks, and post-CVS assessments were conducted immediately after the last session and then 1 and 4 weeks later. The language assessments were administered in the following order; auditory comprehension, repetition, naming, connected speech.

## STIMULATION

**FIGURE 2:**
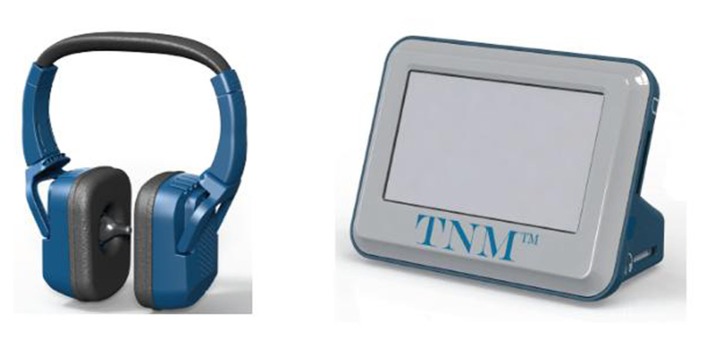
**Illustration of the thermo-modulation device comprising two headphone-mounted ear-probes and an AC powered control unit**.

Caloric vestibular stimulation was administered via a custom-built, experimental device that modulates the temperature of small, thermo-electric, solid-state probes inserted into the right and left external ear canals (see **Figure 2**). The probes are mounted on a headset and are too large to enter the bony portion of the canal, resting instead on the outer fleshy portion. The probes are held in place by first ensuring that the headphones are properly seated on the head (which ensures that the probes are seated within the ear canals) and then fastening a velcro head-strap to prevent further movement. Each probe can be warmed/cooled independently depending on whether unilateral or bilateral stimulation is required, and is controlled with a hand-held unit that both powers the headset and allows the laterality, duration, and temperature range to be regulated. Actual earpiece temperature is monitored by an embedded thermistor (in the tip of the earpiece) which serves as the control point in a PID (proportional/integral/derivative) temperature controller designed to control overshoot. The actual recorded temperatures are recorded on an SD card and can be viewed to confirm temperature compliance. The mode of action is identical to that of conventional caloric irrigators in that heat is conducted back and forth between the external auditory canal to the inner ear via the temporal bone. Unlike caloric irrigators, however, the device allows for physician-defined, time-varying waveform shapes that can be cycled to maximize vestibular response and reduce physiological habituation.

Otoscopic inspection was performed immediately prior to the first session of stimulation to check for excessive cerumen (which may limit temperature transfer) and to confirm that the external ear canal and tympanic membrane were normal in appearance. With the intention of increasing activity in the damaged left hemisphere, CVS was applied to the right ear canal for 30 consecutive days excluding weekends. CVS sessions lasted 20 min during which time probe temperature cycled continuously between 35 and 17°C, achieving approximately eight complete cycles. This gentle transition between warm and cool made the procedure easy to tolerate, and as expected, participants showed no evidence of disorientation or discomfort during stimulation, nodding when asked if the procedure was comfortable. Throughout stimulation, participants sat upright and were not engaged in prolonged conversation or speech or language tasks.

### STATISTICAL APPROACH

Ninety five percent confidence intervals were constructed to determine whether changes from baseline were greater than that which could be attributed to natural variation. For each subtest, upper and lower confidence limits were calculated as two standard errors (SE) above and below the baseline scores, respectively, using the equation SE = SD√1 - ρ where ρ refers to Cronbach’s alpha and where the SD was derived from the responses of an allied sample containing 85 aphasic and 15 elderly normal participants (Jacobson et al., 1999; Goodglass et al., 2001; Reise and Haviland, 2005). Given that each participant showed little change across the two baseline administrations of each subtest, the two baseline scores were combined to produce a more reliable estimate of language ability (Weller, 2007). Adjusted reliability estimates (ρ) were therefore calculated using the Spearman–Brown Prophecy Formula (see Nunnally, 1967). To reduce the likelihood of mistaking experimentally induced recovery with that which was already occuring, 95% confidence intervals were only calculated for those subtests in which the change between the baseline and post-CVS scores was numerically greater than the change between the two baseline scores.

There were two instances in which an average baseline was not calculated; participant 001’s Basic Word Discrimination and participant 003’s Responsive Naming. In both instances, there was a significant difference between the two baseline scores (though this was a much smaller difference than was seen between pre and post-CVS). In these cases it was deemed inappropriate to use an average pre-treatment score and therefore 95% confidence intervals were calculated around the higher of the two baseline measures. In these cases, reliability of the original scale, as opposed to the combined scale, was used.**

To provide broader insight into wellbeing and functional recovery, written testimonials from participants’ relative/carers are presented below alongside the inferential statistics. These testimonials are, of course, anecdotal and subjective, but nevertheless hold corroborative value.

## RESULTS

### PARTICIPANT 001

Boston Diagnostic Aphasia Exam subtest scores are presented in **Table 2**.

**Table 2:** BDAE scores for participant 001.

		Pre1	Pre2	Post1	Post2	Post3
Naming	BNT (15)	2	2	6^*^	7^*^	5^*^
	Colors (4)	0	0	0	0	0
	Animals (12)	3	3	2	4	4
	Actions (12)	2	4	6	4	4
	Tools (12)	2	2	5^*^	6^*^	3
	Responsive naming (20)	1	3	7*	4	2
Connected speech	Cookie theft	3	1	1	1	1
Repetition	Single words (10)	7	7	8	8	7
	Non-sense words (5)	2	3	3	4	2
	Sentences (10)	0	1	0	0	0
Auditory C’hension	Word discrimination (37	21	24	30^*^	30^*^	26
	Commands (15)	6	6	10^*^	9^*^	10^*^
	Complex Ideational (12)	2	2	2	1	0

* Denotes score greater than two standard errors from the baseline mean. Parenthesized values denote maximum score for that subtest. Pre1 and Pre2 = first and second baseline assessment sessions, respectively. Post1, 2, and 3 = assessment sessions conducted immediately, 1 and 4 weeks after CVS, respectively.

Statistically significant improvements from baseline were observed in naming and comprehension. Reliable changes were observed on the BNT short form at all follow-up sessions, category naming of Tools during the immediate and post-CVS week 1 session, and responsive naming at immediate follow-up. For auditory comprehension, reliable changes were observed in the commands sub-test at all follow-up sessions, and for word discrimination at the immediate and post-week 1 follow-up.

The above improvements are echoed in the testimonial below.

#### Testimonial 1 provided by a carer of 001

“*As the carer of XX I wish to share some improvements that have been noticed since she received stimulation at Canterbury University. Her speech is much clearer and she speaks with more confidence. She initiates conversation and is able to let her needs be known. She says short sentences (e.g., Can I have a cup of tea, please?), and has been identifying words and pictures. XX now asks to walk and can walk from the lounge to the kitchen and toilet with the aid of a three pronged stick. XX gets up from the wheelchair and gets herself out of bed into the sitting position. Her right vision seems to have improved. She is also more alert and confident and her facial expressions coincide with what she is trying to express. She clearly understands what is being said to her and expresses/shows appropriate empathy*.”

### PARTICIPANT 002

Boston Diagnostic Aphasia Exam subtest scores are presented in **Table 3**. No statistically significant changes from baseline were observed.

**Table 3:** BDAE scores for participant 002.

		Pre1	Pre2	Post1	Post2	Post3
Naming	BNT (15)	0	0	0	0	0
	Colors (4)	0	0	0	0	0
	Animals (12)	1	0	1	1	0
	Actions (12)	1	1	2	2	0
	Tools (12)	0	0	0	0	0
	Responsive naming (20)	0	0	0	0	0
Connected speech	Cookie theft	4	1	0	1	2
Repetition	Single words (10)	1	1	2	0	0
	Non-sense words (5)	0	0	0	0	0
	Sentences (10)	0	1	0	0	0
Auditory C’hension	Word discrimination (37)	32	29	31	31	32
	Commands** (15)	12	14	14	14	13
	Complex Ideational (12)	6	4	6	6	7

The following testimonial did, however, highlight some potential change.

#### Testimonial 2 provided by 002’s wife

“*Since XX started the treatment, his walking and standing has improved a lot. Some of his speech has come back like ‘goodnight,’ ‘I am starving,’ and ‘I will wash-up’*.”

### PARTICIPANT 003

Boston Diagnostic Aphasia Exam subtest scores are presented in **Table 4**. Statistically significant improvements from baseline were observed on the BNT short form at immediate and week 4 follow-up, on category naming of actions at immediate and week 1 follow-up, and for animals at immediate follow-up. Responsive naming also improved at immediate and week 1 follow-up, and sentence repetition improved at all follow-up sessions.

**Table 4:** BDAE scores for Participant 003.

		Pre1	Pre2	Post1	Post2	Post3
Naming	BNT (15)	5	6	8^*^	4	8^*^
	Colors (4)	2	3	2	3	3
	Animals (12)	4	5	8^*^	6	6
	Actions (12)	5	5	7^*^	7^*^	6
	Tools (12)	3	5	5	7	5
	Responsive naming (20)	3	6	11^*^	10^*^	9
Connected speech	Cookie theft	1	6	4	4	4
Repetition	Single words (10)	6	8	9	8	9
	Non-sense words (5)	3	4	4	4	4
	Sentences (10)	3	4	7^*^	6^*^	6^*^
Auditory C’hension	Word discrimination (37)	37	37	36	36	37
	Commands** (15)	14	14	15	15	14
	Complex Ideational (12)	8	8	9	8	6

* Denotes score greater than two standard errors from the baseline mean.

The above improvements are echoed in the testimonial provided by 003’s wife.

#### Testimonial 3 provided by 003’s wife

“*Since XX started this trial on 13^th^ May 2013, there has been a marked improvement in his speech. Many people (friends or family) over the phone or face to face, whether they see him on a regular basis or from time to time, have noticed a difference in him during this trial period. I feel that maybe a month/6 weeks before the trial began there was a change happening in XX’s speech, but definitely during the trial things have got better. He’s coming out with short sentences and although it’s a struggle sometimes, he gets there in the end. Something else I feel I should mention is two of XX’s speech therapists paid him a visit on 29^th^ May and they both noticed a marked improvement in his speech, after one of them not seeing him for a few months and the other not seeing him for 3 weeks! To sum up, on the whole, XX is definitely speaking more, making a conscious effort to try to make sentences, and I think a little more confident!*”

## DISCUSSION

Two of the three participants improved from baseline on the BNT short form, responsive naming, and category naming. One of these participants also improved on sentence repetition (003), while the other (001) improved on auditory word discrimination and auditory commands. In six of the eight subtests for which statistical change occurred, improvement persisted beyond the immediate assessment to one or both of the 1 and 4 week follow-ups. Together these data give the first tentative support for the idea that CVS can relieve both expressive and receptive elements of aphasia.

We note that recovery patterns diverged across subtests and patients. On the category naming tests, 001 improved on tools while 003 improved on actions and animals. Only 001 improved on the auditory word discrimination and commands while 002 showed no improvement on any test. It is difficult to determine the source of this variation, although category naming is known to vary considerably across aphasics (Goodglass et al., 1966; see also Hillis and Caramazza, 1991), while the failure of 003 to match 001’s improvement on the comprehension tasks partly reflects the fact that his scores were already at or near ceiling during baseline assessment. The failure of 002 to respond is more troubling, though his radiology shows greater temporal-parietal-frontal encroachment than the others, and he also suffered from severe apraxia of speech which resulted in much lower baseline scores.

Perhaps of more concern is the potential impact of natural recovery and placebo. We are reluctant to attribute high importance to natural recovery because all patients were in the sub-acute/chronic phase (i.e., were at least 8 months post-onset). During this time, it would be surprising if substantial changes in language and communication occurred over the course of a 5-week study period. That said, Participant 003 had already showed modest change in the few months leading up to study, but the degree of change shown after enrolment surprised his physicians and carers. It also seems unlikely that any natural recovery would coincide most strongly with the immediate and week 1 follow-ups rather than the later week 4 follow-up. Such a pattern would be more consistent with a placebo effect. However, if the treatment exerted a strong placebo effect then why did only some of the outcome scores change? And why would both participants show change on the BNT and responsive naming tasks, as opposed to more idiosyncratic and divergent patterns? We can find no evidence from elsewhere that these tasks are more susceptible to placebo effects than other BDAE subtests. More generally, sham-controlled studies involving other forms of non-invasive neuro-stimulation report that the placebo effect in aphasic participants tends to be low. For example, Monti et al. (2008) reported that, relative to baseline, cathodal tDCS improved picture naming by 34% while sham led to a 0.4% improvement. Similarly, Barwood et al. (2012) reported that while active rTMS induced an improvement in overall BDAE score of 18.5 points, sham rTMS induced a change of just 0.17.

How might CVS have contributed to the observed recovery? As mentioned above, right-sided stimulation has been shown to increase metabolic activity in left hemisphere language networks (Baker et al., 2010; Hamilton et al., 2010; Fiori et al., 2011; Szaflarski et al., 2011) and also modulates the distal release of acetylcholine and monoamines relevant to language recovery (Horii et al., 1994; Nishiike et al., 2001; Klein and Albert, 2004; Ma et al., 2007). Of particular interest, increased release of noradrenaline and acetylcholine is associated with naming improvement and verbal memory in aphasic patients (Tanaka et al., 1997; Beversdorf et al., 2007), while increased serotonin can exert a positive effect on language recovery, most likely by counteracting depression and anxiety (Laino, 2004). These projection systems are usually characterized as diffuse rather than unilateral which raises the question of whether they are influenced by the side of caloric stimulation? Unfortunately, microdialysis neurotransmitter studies have yet to compare left versus right CVS so the extent to which the recovery seen here arises from non-lateralised mechanisms remains unclear.

On a related note, given that CVS is associated with recovery from a range of other neuropsychological and psychiatric impairments, we are reluctant to attribute the recovery seen in our participants to a language-specific mechanism. More likely is something akin to the domain-general mechanism described by Schiff and Pulver (1999) in which CVS helps engage a thalamic cortico–cortico gating mechanism involved in the reactivation and reintegration of injured cortical areas and/or the recruitment of novel areas. In addition to this, increases in arousal and alertness may enhance susceptibility to placebo effects within the clinical setting.

On a final, more anecdotal point, the testimonials reported above allude to co-morbid improvements in motor function (see Testimonials 2 and 3). Participant 001, who was previously only able to transfer and walk short distances with assistance, was now able to mobilise independently between rooms in the house with the aid of a walking stick. She was able to transfer herself more independently, reporting that this was due to increased control of her right lower limb. Similarly, participant 002 reported an increased control and flexibility in his right lower limb, resulting in more independent movement around the house. 002 still required supervision when climbing the stairs, however he reported that where before this would take up to 10 min, he was now able to complete the task in approximately 5 min. These observations chime with the findings of Sturt and Punt (2013) who recently showed improved postural control in a group of hemi-spatial neglect patients post-CVS, and give reason to assess motor outcome in future studies.

In summary, we provide preliminary evidence that CVS may help relieve both expressive and receptive symptoms of aphasia. A larger-scale, dose-response, sham-controlled study that specifies a broader range of endpoints involving reading, writing, and activities of daily living, would now seem sensible. Coupled with the beneficial effects of CVS on other stroke conditions such as hemi-spatial neglect (Cappa et al., 1987), pain (McGeoch et al., 2008), and hemi-anesthesia (Bottini et al., 2005), the current data also strengthen the growing idea that CVS triggers a generic compensatory response to brain trauma which may be of relevance to a wide variety of neurological conditions. Given the need for many different brain systems to know if the head is upright, moving and if so, in what direction and at what speed, we believe that the therapeutic reach of vestibular stimulation will prove considerable.

## Conflict of Interest Statement

The authors declare that the research was conducted in the absence of any commercial or financial relationships that could be construed as a potential conflict of interest.
